# Therapeutic Implications of Altered Energy Metabolism in Migraine: A State-of-the-Art Review

**DOI:** 10.7759/cureus.8571

**Published:** 2020-06-12

**Authors:** Syed Adeel Hassan, Umar Farooque, Ali S Choudhry, Bharat Pillai, Fahad N Sheikh

**Affiliations:** 1 Neurology, Dow University of Health Sciences, Karachi, PAK; 2 Internal Medicine, Dow University of Health Sciences, Karachi, PAK; 3 Internal Medicine, Lahore Medical and Dental College, Lahore, PAK; 4 Neurology, Amrita Institute of Medical Sciences, Kochi, IND; 5 Pathology, Sahiwal Medical College, Sahiwal, PAK

**Keywords:** migraine, metabolism, metabolic therapy, phonophobia, autonomic disturbance, metabolic abnormalities, photophobia

## Abstract

Currently, the management strategies aimed at the resolution of migraine are pharmacological. Most of these therapies are known to alter the serotonin balance of the brain. Furthermore, therapies blocking the calcitonin gene-related peptide (CGRP) have also proven to be quite effective in their treatments. However, apart from being expensive, these therapies do not influence premonitory and aura symptoms. This suggests an incomplete approach and an inadequate understanding of the migraine pathophysiology. Recent metabolic studies have indicated that migraine should be considered as an adaptive response to the mismatch between the cerebral energy reserves and expenditure. Therefore, understanding the underlying metabolism helps derive possible novel therapeutic modalities for migraines. In this review, we highlight the underlying metabolic abnormalities found in migraine patients. This will form the basis of our evidence-based discussion on metabolic therapeutic options for migraines.

## Introduction and background

Migraine is described as a neurovascular disease that is chronic and debilitating. It is clinically characterized by the onset of unilateral headache, phonophobia, photophobia, and autonomic dysfunction [1]. It was first described as a hypoglycemic headache in the year 1935 [2]. Currently, migraine is known to affect >15% of the population and is regarded as one of the major causes of disability-adjusted life-years [3]. Advances in migraine research have solely addressed neurovascular, vascular and neurotransmission [4]. Therefore, based on these advances, it is usually managed with repurposed therapeutic agents such as beta-blockers, calcium channel blockers, antidepressants, and antipsychotics. Furthermore, recent advances in the understanding of its pathophysiology have yielded therapeutic options such as antibodies, calcitonin gene-related peptide (CGRP) blockers, and neuromodulation strategies [5]. Taking into account the cascade of pathophysiology, the aforementioned treatment options reduce the peripheral and central inputs of nociceptive stimuli into the trigeminovascular system [5].

In 1982, William Amery provided strong evidence linking metabolism in the pathogenesis of migraine [6]. Over the past few decades, multiple clinical studies have indicated that migraine should be considered an energy-deficit syndrome with mitochondrial dysfunction [4]. It is proposed that the concurrent sensory stimuli overload and energy-reserve deficit increase the activation of the trigeminovascular system [4]. The metabolic abnormalities account for upstream disturbances in the pathophysiological cascade of migraines. Therefore, it can be concluded that metabolic abnormalities predispose an individual to migraine attacks [5]. In this review, we will discuss the metabolic abnormalities and prophylactic metabolic therapies for migraines.

## Review

The metabolic imbalance of migraine

Our current understanding of migraine is limited to the activation of the pain-signaling system (trigeminovascular system). Additionally, researchers have not been able to identify a set of common triggers between patients. In the literature, migraine triggers have been classified into nutritional, environmental, behavioral, hormonal, and psychological (Table 1) [1]. In clinical practice, the most common triggers that are encountered include fasting, skipping meals, sleep deprivation, and emotional stress. The common underlying metabolic burdens to these triggers include amplified oxidative stress, impaired mitochondrial energy metabolism, and inefficient glucose metabolism [7-10].

**Table 1:** Trigger factors for migraine

Trigger category	Specific trigger/s
Behavioral	Dehydrations, skipping meals, fasting, exercise, less sleep
Psychological	Stress
Hormonal	Menses, oral contraceptive pills
Environmental	Bright light, blue light, odors, wind, hypoxia, smoke, perfumes containing phthalates
Nutritional	Cured foods, cheese, milk, citrus, monosodium glutamate, aspartame, glucosamine, chocolate, icecream, alcohol, white wine, red wine, banana, coffee

Studies utilizing magnetic resonance spectroscopy have depicted diminished concentrations of various metabolic substrates in the cerebral cortical matter [11-13]. More specifically, increased levels of adenosine diphosphate, decreased levels of organic phosphate, and decreased phosphorylation potential are seen in patients [4]. This implies that oxidative phosphorylation is markedly diminished in migraine patients both during and in between attacks. A direct quantification revealed a 16% decrease in adenosine triphosphate (ATP) levels in the brains of migraine patients [14]. The quantification supports the correlation between the hypometabolic state and attack frequency. Magnesium is considered a vital cofactor in the synthesis of ATP. Therefore, any reduction in magnesium levels can halt the production of ATP. In a study conducted by Lodi et al., free magnesium levels were deemed insufficient in the occipital lobes of migraine patients [12]. Therefore, the lowest levels of ATP are noticed in patients who suffer from severe and more frequent migraine attacks. These findings suggest that the disease is more severe in patients with profound metabolic disturbances.

Abnormal sensory processing demand leads to neuronal overactivation in migraine. This further warrants the need for higher energy demand. Several studies have shown that lactate levels are elevated in migraine patients with visual aura [4]. However, cerebral lactate levels are normal in patients without an aura [4]. Due to the high variability between studies, a strong association with lactate levels could not be established. In the brain, astrocyte activation leads to energy disposal to the neurons. This is achieved by the astrocyte-neuronal lactate shuttle. Under physiological stimulation, levels of cortical lactate are known to increase. Therefore, in the absence of a physiological stimulus (migraine), increased lactate levels can be considered pathological [4]. It has also been suggested that elevated levels of free fatty acids and ketone bodies have been detected before a migraine attack [1]. An elevation in concentrations of growth hormone, glycerol, and cortisol are also seen during an attack [1]. Furthermore, the ratio of beta-hydroxybutyrate to acetoacetate is also increased. However, a vital point to note is that the insulin levels were deemed low [1]. This suggests a typical picture of a metabolic response seen during stress. The increase in ketogenesis and lipolysis can be thought of as the body's effort to counter the energy deficit and restore cerebral energy homeostasis.

In terms of glucose metabolism, various studies have reported a significant degree of insulin resistance and glucose tolerance [15]. Therefore, higher levels of insulin in migraine patients help us predict reduced insulin sensitivity. Insulin normally functions to promote the uptake of glucose from the bloodstream via insulin-dependent glucose transporter type 4 into peripheral tissues such as adipose tissue and muscles. Furthermore, it is important to remember that the brain is an insulin-independent organ. The glucose transporter type 1 receptor transports glucose across the membranes of astrocytes, oligodendroglia, and the blood-brain barrier [4]. The reduced insulin sensitivity can be regarded as an adaptive response to allow more glucose to be available for the brain. As a consequence, the energy supply of the brain increases. Once the migraine attack ensues, patients tend to avoid the trigger stimuli and consequently rest. This reduces the abnormal sensory stimulation input and restores the metabolic disequilibrium.

A generalized metabolic dysfunction occurs due to the reduced function of the respiratory chain enzymes. The affected enzymes include monoamine oxidase, succinate dehydrogenase, nicotinamide adenine dinucleotide dehydrogenase, cyclooxygenase, and citrate synthetase [4]. These enzymes are encoded by the mitochondrial deoxyribonucleic acid (mtDNA) and are more susceptible to oxidative stress [4]. Patients with migraine have shown increased oxidative stress or antioxidant capacity. This is demonstrated by the reduced activity of superoxide dismutase activity in such patients [4]. Several studies have proposed that increased levels of heavy metals account for oxidative stress. Welch et al. reported the presence of iron deposits in the brain stem of migraine patients [16]. Furthermore, these deposits were proportional to the disease duration [16]. The metabolic abnormalities can also be explained by mutations in the genes encoding metabolic enzymes in both mitochondrial and nuclear DNA [5].

Migraine metabolism and pathophysiology

Activation of Hypothalamus and Brainstem

In the premonitory phase, the initiation of the migraine attack is carried out by the activation of the hypothalamus. The exact activating factor remains unknown. However, the hypothalamus possesses the ability to sense the metabolic imbalance via chemosensitive neurons [4]. These neurons form an extensive network that stretches from the thalamus to the brainstem. Therefore, any metabolic change will activate the chemosensitive neurons, and initiate a migraine attack [4].

Cortical Spreading Depolarization

Cortical spreading depolarization (CSD) is a self-propagating wave of depolarization responsible for the aura experienced in migraine [4]. The spreading depolarization leads to significant water, ionic, and neurotransmitter fluxes [17]. From our current understanding, the pharmacological modulation of CSD is well-known [17]. Changes in metabolic factors can also influence susceptibility to CSD [4]. Hoffmann et al. suggested that the underlying glycemic state and cerebral glucose levels can modulate CSD in vivo [17]. Experimental human studies have noted that there is a rapid increase in glucose utilization in CSD [17]. This leads to markedly decreased levels of glucose within cells. It has been proposed that hyperglycemia prevents the onset of CSD [4,17]. Whereas, hypoglycemia is known to increase susceptibility to CSD [4,17]. Hyperglycemia acts as a buffer to quickly restore the depleted cellular glucose levels during CSD [17]. This prevents the depletion of intracellular glucose concentrations to maintain ATP production [17]. Therefore, in the presence of adequate energy, the ATP-dependent cellular pumps prevent intracellular potassium accumulation; thus preventing the cell from reaching the firing threshold for CSD [17]. Furthermore, hyperglycemia may also depress membrane excitability by increasing lactate production (acidic pH) [18]. Whereas in hypoglycemia, the absolute refractory period of cells is extended [17]. In effect, this increases the overall duration of depolarization and prolongs the CSD [17].

The reduction of local oxygen levels (hypoxia) can also predispose to CSD [19]. Takano et al. demonstrated that CSD is associated with massive neuronal swelling, tissue hypoxia, and temporary loss of dendritic spines [19]. The tissue hypoxia is not deemed due to hypoperfusion. Instead, an increase in the local blood flow during CSD is noted. Therefore, the drop in tissue oxygen levels could be due to excessive elevations in oxygen consumption when compared to its delivery. Furthermore, analysis of their data also proposed that increasing the oxygen availability shortens the duration of CSD and repletes the diminished redox potential (levels of nicotinamide adenine dinucleotide). [19].

Calcitonin Gene-Related Peptide (CGRP) and Trigeminovascular System

Our current understanding suggests that the triggers of migraines result in the dilation of cranial blood vessels [20,21]. This results in the overstimulation and activation of perivascular trigeminal sensory nerves [21]. The activation of the trigeminovascular system permits the transmission of pain stimuli to the higher centers [21]. Furthermore, as a consequence, vasoactive peptides such as CGRP and substance P are released from the trigeminal nerves [21]. Cyclically, these vasoactive peptides further activate trigeminal sensory nerves and mediate the release of more vasoactive peptides. As the cycle progresses, the initially stimulated areas of the brain become sensitized [21]. This results in increased sensitivity to surrounding stimuli and worsening of headache [21]. CGRP is also hypothesized to be involved in the cerebral vessel dilation, dural vessel dilation, inflammatory mediator release from mast cells, and transmission of nociceptive input from the cranial blood vessels to the processing centers of the brain [21]. The result is the development of neurogenic inflammation by mediating vasodilation, blood vessel leakage, and mast cell degranulation [21]. 

Lisicki et al. reported an increased neuronal activation-to-resting glucose uptake ratio in migraine patients between attacks [22]. This suggests that the brain has a concomitantly reduced supply and requirement of energy substrates. The association between altered bioenergetics and trigeminovascular activation can be explained by the activation of a certain channel. Pannexin is a large neuronal channel that functions as a sensor of cortical homeostasis [4]. Under conditions of stress, these channels activate and form a complex with P2X7 receptor (ligand-gated ion channel) [4]. The resulting biochemical cascade accounts for the activation of the trigeminovascular system and CGRP release [4]. The reduction of neuronal metabolic substrates results in the activation of pannexin 1 channels [4]. Therefore, the biochemical cascade occurring due to the activation of pannexin channels can provide an association between energy imbalance in the cortical structures and migraine attacks [4].

Metabolic treatment options for migraine

Ubiquinone

Ubiquinone, or coenzyme-Q10 (CoQ10), is an integral part of the electron transport chain (ETC) located in the inner mitochondrial membrane. It functions as a cofactor in the ETC and possesses antioxidant properties [4,5]. Therefore, the administration of CoQ10 in migraine patients could improve the overall capacity of cells to deal with the overwhelming oxidative burden. It also allows for the rectification of impaired mitochondrial energy metabolism in between attacks. Dahri et al. demonstrated that the supplementation of CoQ10 results in a significant reduction in the levels of CGRP (p = .011) and tumor necrosis factor-α (p = .044) [23]. However, no reduction in the levels of interleukin-6 and interleukin-10 was noted [23]. Furthermore, clinically significant improvement was noted in terms of the frequency (p = .018), severity (p = .001), and duration (p = .012) of migraine attacks [23].

Hershey et al. conducted a study to assess the prevalence of CoQ10 deficiency in pediatric and adolescent migraine patients [24]. Furthermore, they closely followed the effects of CoQ10 supplementation on headache improvement as well as headache disability [24]. The results indicated that 32.9% of patients were below the normal reference range [24]. These patients were started on 3 mg/kg/day of CoQ10 via capsules [24]. Patients returning for timely follow-up were noted to have CoQ10 level improved to 1.20 +/- 0.59 microgram/mL (p < .0001) [24]. They also noticed an improvement in headache frequency from 19.2 +/- 10.0 to 12.5 +/- 10.8 (p < .001) [24]. Pediatric Migraine Disability Assessment Scores also improved from 47.4 +/- 50.6 to 22.8 +/- 30.6 (p < .001) [24]. These results suggest that CoQ10 deficiency can be common in pediatric and adolescent migraine patients. Rectification of CoQ10 deficiency can result in overall clinical improvement. Therefore, it is recommended that CoQ10-deficient patients can be started on CoQ10 supplementation [24].

In an open-label trial, the efficacy of CoQ10 as a preventative migraine treatment was assessed [25]. CoQ10 (150 mg/day) was administered to 32 migraine patients. After three months of therapy, It was noted that the average number of migraine days dropped from 7.34 to 2.95 (p < .0001) [25]. A total of 61.3% of patients experienced a ≥50% reduction in the number of migraine days [25]. A reduction of 13.1% in migraine frequency was noted at the end of one month of therapy [25]. The migraine frequency further dropped by 55.3% after three months of therapy [25]. Furthermore, the mean attack frequency significantly decreased from 4.85 to 2.81 attacks (p < .001) [25]. In addition to the aforementioned findings, no adverse effects of CoQ10 therapy were noted. In another randomized control trial, the efficacy of a liquid suspension of CoQ10 at double doses was investigated. The study concluded that CoQ10 is superior to placebo for attack frequency, headache days, and days with nausea in the third treatment month [25]. Of the 42 patients in the study, only one developed cutaneous allergy [26]. This indicates a good tolerance to therapy with minimal adverse effects. More recently, an open-label, add-on, controlled trial was conducted to determine the effect of CoQ10 supplementation on the adult population. The administration of 100 mg of CoQ10 daily was shown to reduce the number of attacks per month and headache severity [27]. Comparable to other studies, no adverse events to CoQ10 therapy were noted [27]. 

In summary, CoQ10 deficiency is common among pediatric and adolescent migraine patients. Efforts must be made to determine and rectify the level of CoQ10 deficiency. Based on the encouraging findings of various clinical studies, CoQ10 as a preventative therapy is well tolerated with minimal adverse effects. Furthermore, it possesses the ability to reduce the severity, frequency, and duration of migraines.

Riboflavin

Riboflavin is the precursor of flavin mononucleotide and flavin adenine dinucleotide. The flavin nucleotides are considered immensely necessary for the activity of various flavoenzymes (electron carries) of the ETC [4,5]. Therefore, riboflavin plays a crucial role in the metabolism of various metabolic substrates such as carbohydrates, fats, and proteins [4]. It allows the recycling of oxidized glutathione [4]. Therefore, it carries significant antioxidative capabilities. Mechanistically, it reduces brain calcium levels and microsomal lipid peroxidation [28]. Furthermore, they enhance the antioxidative capabilities in the brain by increasing the levels of antioxidants [28]. Increased cytosolic calcium levels result in excessive mitochondrial calcium uptake [28]. As a consequence, mitochondrial membrane depolarization and stimulation of the Krebs cycle ensue [28]. The increased intramitochondrial metabolism results in the subsequent depletion of pyridine nucleotides [28]. High cytosolic-free calcium is also known to enhance the production of reactive oxygen species (ROS) [28]. Riboflavin possesses the potential to reduce the production of ROS and cytosolic calcium levels [28]. This is achieved via modulation of voltage-gated calcium channels and transient receptor channels [28]. In summary, riboflavin carries a significant neuroprotective role by reducing oxidative stress, mitochondrial dysregulation, and neuroinflammation.

In an open-label study conducted by Boehnke et al., patients were administered riboflavin capsules (400 mg) daily for six months [29]. It was observed that the headache frequency decreased from four days per month to two days per month at three and six months, respectively (p < .05) [29]. However, no significant changes in headache duration or headache intensity was noted [29]. Treatment with riboflavin also allowed for the reduced consumption of abortive medications [29]. A single-blind, randomized clinical trial comparing riboflavin and sodium valproate yielded similar results in the reduction of migraine attacks. A similar reduction in the severity, frequency, and duration of headaches was noted with both medications [30]. Furthermore, significantly fewer adverse effects were reported with the use of riboflavin (p = .005) [30]. This finding warrants the use of riboflavin in migraine patients who experience adverse effects on sodium valproate therapy.

Clinical studies have also been conducted to determine the dosage and efficacy of riboflavin administration in pediatric and adolescent migraine patients. In a retrospective study of 41 patients, pediatric and adolescent migraine patients were administered riboflavin at a single daily dose of 200 or 400 mg [30]. Patients reported decreased headache intensity and attack frequency during the treatment (p < .01) [30]. During follow-up, 68.4% of cases were responders with a frequency reduction of at least 50%, whereas attack intensities were reportedly reduced by 21% [30]. Furthermore, a study conducted by Bruijn et al. helped to establish the dose of riboflavin required for prophylactic prevention of migraines in pediatric patients. Administration of riboflavin at a dose of 50 mg/day did not result in a significant reduction in the frequency of migraine attacks (p = .44) [31]. Based on the findings of these clinical studies, riboflavin is efficacious for migraine prevention in adults and children at a daily dose of 200-400 mg. Low-dose riboflavin (50mg) does not seem to promote migraine prevention and should not be prescribed for migraine prevention.

Pharmacogenetic studies have also helped delineate the relationship between therapeutic response to riboflavin and mtDNA haplogroups [32]. Migraine patients possessing the haplogroup H mtDNA did not respond effectively to the treatment with riboflavin [32]. Likewise, patients belonging to the non-haplogroup H mtDNA responded efficiently to riboflavin prophylaxis [32]. The exact reason for this association remains unknown. One possible hypothesis suggests that mtDNA haplogroups influence the activity of ETC complexes [32]. It has been proposed that haplogroup H is associated with a 20% decrease in the activity of ETC complexes 1 and 4 [32].

Lipoic Acid

Lipoic acid is a water- and fat-soluble cofactor for enzymes in the Krebs cycle. It functions as a scavenger to remove ROS and indirectly chelate metal ions [4]. In doing so, it reduces oxidative stress and is classified as an antioxidant [4]. It is also known to increase cerebral energy reserves [33]. Furthermore, it also improves the insulin resistance seen in migraine patients [34].

In a study conducted by Cavestro et al., 32 patients were administered 400 mg of lipoic acid twice daily for six months [34]. A reduction in the frequency of attacks by at least 50% was noted in 53% of the patients at two months (95% CI: 0.36-0.70), 56% at four months (95% CI: 0.39-0.73), and 69% at six months (95% CI: 0.53-0.85) [34]. The incidence of attack ratios at six months decreased from 11 attacks (10-12) to five attacks (4-6) [34]. The number of days of abortive treatment reduced from 7.7 days (6.8-8.7) at baseline to 5.4 days (4.6-6.2) at two months, 5.3 days (4.5-6.1) at four months, and 4.3 days (3.6-5.0) at six months [34]. Therefore, the results obtained in this study provide evidence of a reduction in the number of attacks and treatment days in migraine patients with insulin resistance [34].

Ali et al. conducted an open-label study, in which the combined efficacy of topiramate (50 mg/day) and lipoic acid (300 mg/day) for migraine prophylaxis was assessed [35]. Combined topiramate/lipoic acid therapy was deemed more effective than monotherapy with each medication. The combined therapy reduced the mean monthly migraine frequency from 5.86 ± 1.2 to 2.6 ± 0.98 (p ⩽ 0.05) [35]. Assessment of mean monthly migraine days revealed a decrease from 12.32 ± 1.85 days to 5.74 ± 1.1 days [35]. Furthermore, the responder rate was noted at 85% in patients receiving combined therapy [35]. In patients receiving monotherapy, the responder rate was noted at 30% with topiramate monotherapy and 20% with lipoic acid monotherapy [35]. The patients receiving topiramate (50 mg) monotherapy also reported an increased incidence of adverse events, whereas combined therapy was well tolerated [35]. In conclusion, lipoic acid can be an efficient add-on therapy with topiramate.

L-Carnitine

The carnitine shuttle/transporter mediates the entry of fatty acids into the mitochondria via the inner mitochondrial membrane. In the mitochondria, fatty acids undergo beta-oxidation to yield acetyl coenzyme A for energy production. Esfanjani et al. conducted a study in which 133 patients were randomly assigned into three groups [36]. These groups received magnesium oxide (500 mg/day), L-carnitine (500 mg/day), and Mg-L-carnitine (500 mg/day magnesium and 500 mg/day L-carnitine), respectively, in addition to the conventional therapies [36]. At 12 weeks, it was noted that migraine indicators such as migraine attacks per month, migraine days per month, and headache severity showed significant reductions in all three groups (p < .05). [36]. However, more reductions were observed in the group receiving magnesium oxide [36]. Conversely, when compared to placebo, Hagen et al. provided no statistically significant difference and benefit from acetyl-l-carnitine administration (3 g/day) [37]. Hajihashemi et al. studied the efficacy of concurrent administration of CoQ10 (30 mg/day) and L-carnitine (500 mg/day) supplementation [38]. The results indicated a significant reduction in the levels of lactate (-2.28 mg/dl, 95% CI: -3.65, -0.90; p = .002), severity (-3.03, 95% CI: -3.65, -2.40; p ≤ .001), duration (-7.67, 95% CI: -11.47, -3.90; p ≤ .001), and frequency (-5.42, 95% CI: -7.31, -3.53; p ≤ .001) after eight weeks [38]. These findings suggest that co-administration of CoQ10/L-carnitine supplements results in the improvement of severity, duration, and frequency of migraine attacks [38]. Furthermore, this combined therapy can also help reduce serum lactate levels in migraine patients [38].

Magnesium

Magnesium is an essential cofactor for at least 300 enzymes in energy metabolism [4]. Due to its widespread requirement, it is a vital component of various metabolic pathways. Several studies have indicated reduced blood levels of magnesium in migraine patients [4]. This deficiency can easily disrupt widespread metabolic reactions and creates an underlying metabolic imbalance. Administration of intravenous magnesium has been known to have a modest benefit in acute migraine attacks [4]. A reduction in the attack frequency and intensity can be achieved via oral magnesium administration [4]. 

Dietary Ketogenesis and Pharmacological Ketogenesis

Ketogenic (Atkins) diet requires the modification of dietary intake to consume large amounts of fats, proteins, and minimal carbohydrates. It mimics a state of fasting and switches the energy substrate required for the brain [4]. Central to the process is the production of endogenous ketone bodies such as acetoacetate, beta-hydroxybutyrate, and acetone. They can easily penetrate the blood-brain barrier and serve as an alternative means of fuel for energy synthesis during prolonged starvation, hypoglycemia, and increased neuronal activity [5]. Ketone bodies can block ATP-sensitive potassium channels and reduce neuronal reactivity [4,5]. This forms the basis of utilizing ketogenesis as a therapeutic strategy in migraine prophylaxis.

The use of exogenous ketone supplementation can form the basis of pharmacological ketogenesis. Exogenous ketones (beta-hydroxybutyrate) can restore Krebs cycle intermediates in a process known as anaplerosis [5]. In an open-label study, triheptanoin (a ketone body donor) was able to improve the bioenergetic profile in the brain [39]. This was achieved by increasing the brain inorganic phosphate to phosphocreatine ratios in the brain of Huntington disease patients [39]. However, dietary ketone donors notoriously cause poor oral gastrointestinal tolerance [5]. Therefore, exogenous ketone supplementation has the potential to become a preventative therapeutic option for migraines.

Strategies to improve mitochondrial dysfunction and energy imbalance

Based on the above discussion, we recommend the following strategies to address mitochondrial dysfunction and energy imbalance in migraine patients:

Firstly, one must determine the patients' micronutrient status using laboratory tests. Any deficiency must be rectified by the prescription of appropriate supplementations. Prescriptions can include vitamins, minerals, and trace minerals [4]. This will ensure that the optimal concentrations of nutrients are available for adequate mitochondrial function [4].

Secondly, one must adopt strategies that will help reduce oxidative stress and enhance antioxidant capabilities. These include the elimination of high glycemic index foods, processed foods, alcohol, hormone-based contraception, and the use of green or blue filtering glasses [4]. Antioxidants such as lipoic acid, polyphenols, CoQ10, and beta-hydroxybutyrate salts must be added to the diet [4]. Patients should also be counseled about making lifestyle changes.

Thirdly, if the patient presentation suggests glucose intolerance, an oral glucose tolerance test must be performed. This test must also be conducted if the patient's family history is positive for glucose intolerance [4]. Migraine patients are likely to benefit from the stabilization of blood glucose [4].

Lastly, one should consider providing an alternative source of energy for the brain. This can be achieved by adopting a ketogenic diet or exogenous ketogenic substances [4]. The options for the latter subtype include ketone body salts and medium-chain triglycerides [4]. 

## Conclusions

The underlying metabolic alterations imply that migraine is an adaptive conserving response. It helps maintain cerebral energy homeostasis and further decreases oxidative stress. From our review of the metabolic therapeutic options, it is quite evident that these are inexpensive, well-tolerated, and well-supported options for migraine prophylaxis. Of all the options discussed, riboflavin possesses the most favorable profile. Therefore, it should be implemented as a first-line agent in adult patients with another abortive/preventative drug. In pediatric patients, riboflavin and CoQ10 can be considered for first-line use. Better efficacy and tolerance to prophylactic therapy can be achieved by the coadministration of lipoic acid and topiramate. In patients with underlying insulin resistance, lipoic acid provides a reduction in the number of attacks and treatment days. Coadministration of CoQ10 and L-carnitine should be considered to provide a reduction in the severity, duration, and frequency of migraine attacks. Currently, studies linking biological data and the effects of metabolic treatments are insufficient. Better characterization of the patient's genotype/phenotype is also required.
